# Correlation between ultrasonographic findings and symptoms of knee osteoarthritis

**DOI:** 10.20407/fmj.2018-013

**Published:** 2019-02-06

**Authors:** Osamu Niwa, Shinichi Kato, Nobuki Terada

**Affiliations:** Department of Orthopaedic Surgery, Restorative Medicine of Neuro-Musculoskeletal System, Fujita Health University Bantane Hospital, Nagoya, Aichi, Japan

**Keywords:** Knee osteoarthritis, Japanese Knee Osteoarthritis Measure, Ultrasonography, Radiography

## Abstract

**Objectives:**

Knee osteoarthritis (OA) is mainly diagnosed by clinical and radiographic findings. The aim of this study was to evaluate the correlation between ultrasonography (US) findings during flexion and knee loading and symptoms of knee OA.

**Methods:**

We studied 33 knees with OA in 21 patients. Using US, the medial meniscal protrusion was measured at the midpoint of the medial joint space with the patient standing and the knee in maximum extension and flexion. With the knee in extension, the thickness of the synovial membrane at the suprapatellar area and the size of the osteophytes at the medial joint space were measured. Radiography was performed to determine the Kellgren-Lawrence (K-L) scores. The correlations between US findings and the visual analog scale (VAS) score, Japanese Knee Osteoarthritis Measure (JKOM) score, K-L score, and range of motion (ROM) were analyzed.

**Results:**

Medial meniscal protrusion was significantly correlated with K-L score and ROM limitation. Synovial membrane thickness was also significantly correlated with the total JKOM and usual activity scores. There was no correlation between the VAS scores and US findings. Multigroup comparisons of the patients’ positions during US did not reveal significant intergroup differences.

**Conclusions:**

US was able to detect a change in medial meniscal protrusion during knee flexion and loading. Although medial meniscal protrusion was not correlated with pain, it was related to structural changes of the knee, similar to radiographic findings. Synovial membrane thickness detected by US correlated with pain and a disturbance in the usual activity of patients with OA.

## Introduction

Osteoarthritis (OA) of the knee is one of the most common musculoskeletal diseases, and pain and range of motion (ROM) disturbance are considered the main abnormal findings.^1^ OA symptoms are multifactorial, and the mechanism of the disease is not completely known. In early knee OA, the clinical symptoms related to the degree of synovitis are caused by cytokines. However, in severe knee OA, cytokine levels are decreased, and symptoms are related to biomechanical factors, including meniscal failure and joint instability caused by ligament abnormalities.^2,3^ However, a recent systematic review by Yusuf et al.^4^ showed no relationship between pain and cartilage defects, osteophytes, meniscal lesions, ligamentous abnormalities, subchondral cysts, and bone attrition on magnetic resonance imaging (MRI), and that bone marrow lesions and effusion/synovitis on MRI may be the cause of the pain in knee OA.

The diagnosis of OA is based mainly on clinical findings, but imaging methods are useful for identifying the structures involved in the pathological process, prognosis, and follow-up.^5^ Historically, the first imaging method used for the diagnosis of OA was radiography, which evaluates the bone structure. It reveals marginal osteophytes, narrowing of the joint space, bone cysts, and subchondral sclerosis. MRI is very useful to evaluate joint changes in OA, including osteophytes, bone marrow lesions, subchondral cysts, bone attrition, meniscal tears, ligament abnormalities, synovial thickening, joint effusion, intra-articular loose bodies, and periarticular cysts^6^; however, it cannot be employed as a routine examination owing to its high cost and relatively low availability. Computed tomography (CT) is useful for evaluating bone changes, but it is rarely used for OA in clinical practice.^7^

Ultrasonography (US) is relatively easy to perform and is a non-invasive imaging technique that produces minimal discomfort. It can assess intra-articular abnormalities in knee OA^8,9^ reliably and is helpful in its diagnosis.^10^ Furthermore, US can be used under various conditions such as joint flexion and loading that cannot be evaluated by other modalities. Many studies have been performed to establish the possible correlations between US findings and knee OA symptoms.^8,11,12^ A recent study indicated that US is a useful and reliable method for identifying knee osteophytes, medial meniscal protrusion, and morphological changes in the cartilage in the medial femoral condyle. US detects osteophytes and medial meniscal protrusion better than conventional radiography.^10,13^ Kijima et al.^14^ reported a correlation between pain and medial meniscus protrusion in 38 patients with knee OA. However, no study has reported on US findings in a knee under flexion or loading. In this study, we evaluated the correlation between knee OA symptoms and US findings in a flexed or loaded knee.

## Methods

### Patients

From July to December 2016, we studied 33 knees with OA (21 patients) that were diagnosed based on clinical and radiographic findings. This study was approved by the ethics committee of Fujita Health University (approval no. HM16-075). All patients were fully informed and agreed to the concept and aim of this study. All examinations (clinical, US, and radiographic) were performed on the same day by the same orthopedic physician.

### Clinical examination

The ROM was measured in active flexion and extension. The Japanese Knee Osteoarthritis Measure (JKOM) and visual analog scale (VAS) scores were calculated for each patient. The JKOM is a five-part questionnaire: part 1 is synonymous with the VAS score; part 2 assesses pain and stiffness; part 3 assesses the state of daily life; part 4 assesses the usual activity; part 5 assesses the influence of health conditions. Each of the 25 questions from parts 2 to 5 is graded from 0 to 4 according to severity. We defined the total score from parts 2 to 5 as the total JKOM score.

### Ultrasonography

**Figure 1:**
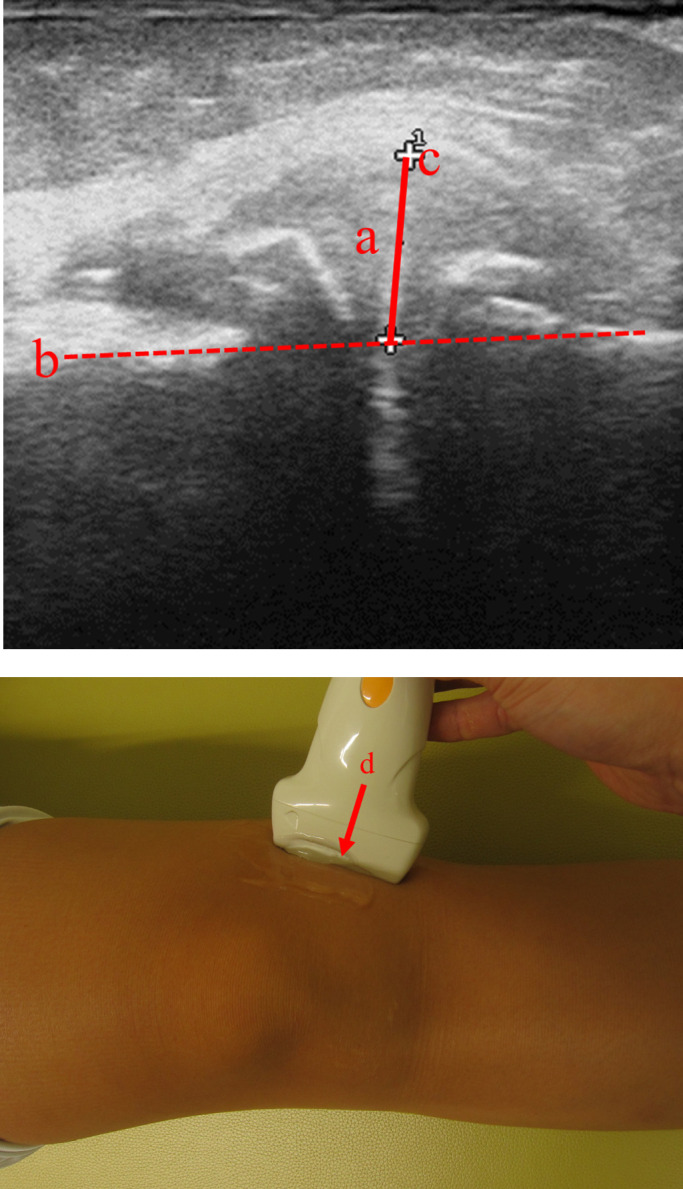
Longitudinal scan of the medial compartment. (a) Medial meniscal protrusion. (b) Bone contour line. (c) Outer edge of the meniscus. (d) Midpoint of the medial joint space.

**Figure 2:**
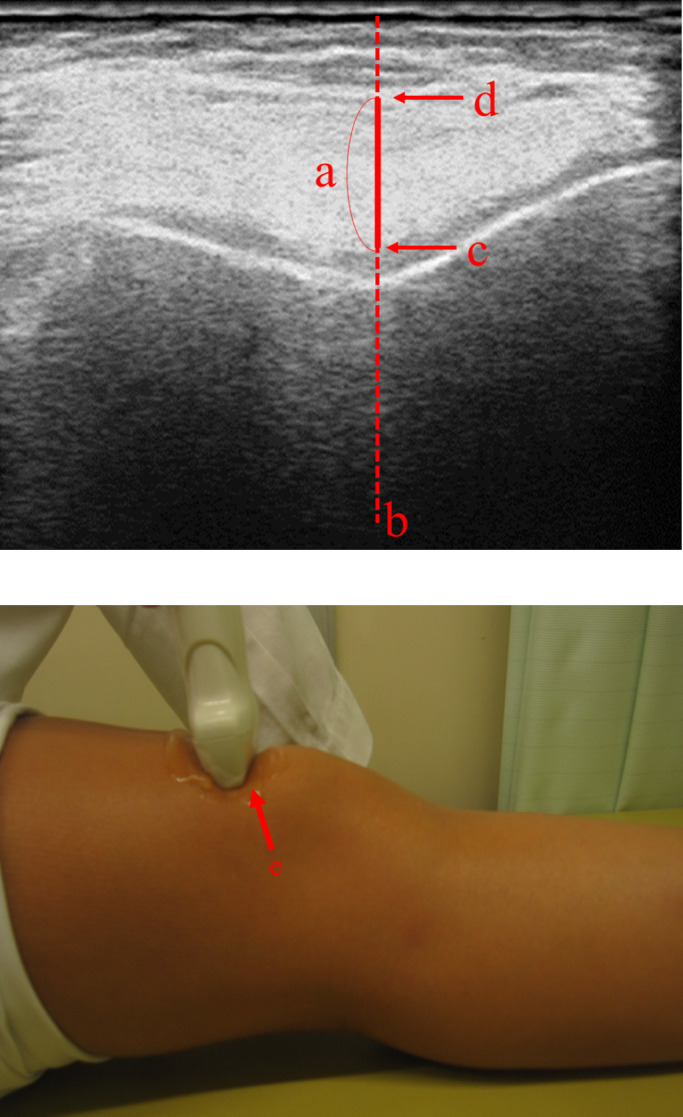
Transverse plane with the echo probe in contact with the proximal aspect of the patella. (a) Synovial membrane thickness. (b) Perpendicular line of the central femoral bone. (c) Cartilage surface. (d) Synovial surface. (e) Proximal aspect of the patella.

US was performed using a MyLab25 (Hitachi, Ltd., Chiba, Japan) ultrasound imaging system with a 12-MHz linear transducer. The length of the medial meniscal protrusion was measured in three positions: supine with maximum knee extension; supine with maximum knee flexion; standing with full loading of the knee. It was measured on the midpoint of the medial joint space and was used to define the distance on the joint line between the outer edge of the meniscus and the bone contour line during a longitudinal scan (Figure 1). The size of the femoral osteophyte was measured on the midpoint of the medial joint space while in the supine position with maximum knee extension (Figure 1). Synovial membrane thickness, which was measured in the transverse plane with the echo probe in contact with the proximal aspect of the patella while in the supine position with maximum knee extension, was defined as the distance on the perpendicular line of the central femoral bone between the cartilage surface and synovial membrane surface (Figure 2).

### Radiography

Anteroposterior and lateral X-rays were obtained in the standing and supine positions, respectively. The Kellgren-Lawrence (K-L) score^15^ was used to classify knee OA radiographic findings as grades 0–4: grade 0=normal; grade 1=doubtful narrowing of the joint space, with possible osteophyte development; grade 2=definite osteophytes, but absent or questionable narrowing of the joint space; grade 3=moderate osteophytes, with definite narrowing, some sclerosis, and possible joint deformity; grade 4=large osteophytes, with marked narrowing, severe sclerosis, and definite joint deformity.

### Statistical analysis

The correlation between US findings and the K-L score, ROM, VAS score, and JKOM score were statistically evaluated using the Spearman correlation coefficient. The population was approximated by a t-distribution. Multigroup comparisons of the patients’ positions during US (extension, flexion, and loading) were analyzed using one-way analysis of variance (ANOVA). P<0.05 was considered statistically significant. Statistical analyses were performed using Windows 10 and Excel 2013 (Microsoft, Redmond, WA, USA) and Statflex version 6.0 (Artec Co. Ltd., Osaka, Japan).

## Results

The characteristics of the study population are presented in Table 1. The size of the femoral osteophyte was significantly correlated with the length of the medial meniscal protrusion during extension (P=0.01398), flexion (P=0.01103), and standing (P=0.00968). The correlations between US findings and K-L score, ROM, VAS score, and JKOM score are presented in Table 2. The length of the medial meniscal protrusion in all patient positions significantly correlated with the K-L score and flexion ROM. The size of the femoral osteophytes did not correlate with K-L score, ROM, VAS score, or JKOM score. The VAS score did not correlate with US findings. The total JKOM score and usual activity score significantly correlated with synovial membrane thickness.

Multigroup comparisons of the patients’ positions during US (extension, flexion, standing) using one-way ANOVA (F=0.1705, P=0.1795) of the length of medial meniscal protrusion showed no significant difference between the patients’ positions during US.

## Discussion

Knee OA symptoms are multifactorial; therefore, it is necessary to evaluate the symptoms of patients. The Western Ontario and McMaster Universities Osteoarthritis Index (WOMAC) has been used internationally as a patient-based measurement of the disease. No authorized Japanese edition of the WOMAC exists, but a translated version is used in Japan. Meanwhile, the Japanese Orthopaedic Association and the Japanese Society for Musculoskeletal Rehabilitation developed the JKOM score.

This study demonstrated that the VAS score did not correlate with US findings, but the total JKOM and usual activity scores correlated with synovial membrane thickness, and ROM correlated with the length of the medial meniscal protrusion in all positions.

Our result demonstrated no correlation between the length of the medial meniscal protrusion and VAS and JKOM scores. However, Kijima et al.^14^ reported a correlation between pain and medial meniscus protrusion in 38 patients with knee OA. Notably, we measured the length of the medial meniscal protrusion differently from Kijima et al., as they did not consider the presence of osteophytes. Podlipska et al.^13^ used the scale proposed by Koski et al.^16^ to evaluate osteophytes and the distance from the bone contour (Figure 1) to determine meniscal protrusion, which highlighted the additional value of US compared with radiography for knee OA management. One drawback of the study by Podlipska et al. was that they did not correlate these findings with pain. Chan et al.^7^ positively correlated the presence of medial osteophytes and meniscal protrusion with pain when climbing stairs but not with pain when walking. In our study, we evaluated the global level of pain and used the JKOM score because we did not focus on specific physical activities.

We did not observe a significant difference between the patients’ positions during US with regard to medial meniscal protrusion. Yanagisawa et al.^10^ reported that the length of the medial meniscal protrusion was significantly longer in the loaded position in 131 knees. In our study, the mean length of the medial meniscal protrusion was 7.44±3.42 mm in the extended position and 8.52±3.94 mm in the loaded position. We speculate that the difference between the results of Yanagisawa et al. and our results could be mainly due to the difference in the size of the population. If more patients were enrolled in this study, the difference between the patients’ positions during US with regard to medial meniscal protrusion may have been significant.

In severe knee OA, patients often complain of loading pain and maximum flexion pain, and we hypothesized that the medial meniscal protrusion during loading and flexion would correlate with knee OA pain in these positions. However, there was no significant difference in the patients’ position and the correlation between the medial meniscal protrusion and VAS and JKOM scores. Rather, the medial meniscal protrusion correlated with ROM limitation and the K-L score, which revealed that the medial meniscal protrusion was not correlated with pain, but with joint structural changes, similar to the radiographic findings. These results suggest the possibility of using US as a substitute for radiographic evaluation.

Meanwhile, we found that the total JKOM and usual activity scores were correlated with synovial membrane thickness. This reflected synovitis-related pain and disturbance in the usual activity of OA patients. This result was shown by our study, which mostly included patients with early-stage OA.

Some limitations of our study should be considered. US is a reliable, real-time, and operator-dependent imaging technique. An important limitation of our study was the lack of MRI findings. Additionally, the psychological factors that could interfere with pain in our patients were not evaluated. Furthermore, the number of study participants with severe OA was relatively small. Thus, the correlation of knee OA symptoms and US findings remains unclear and controversial. Larger studies are required to further clarify this issue.

## Figures and Tables

**Table1:** Characteristics of the study population

Patients, n (female/male)	21 (18/3)
Examined knees, n	33
Age, mean±SD (years)	73.45±8.95
Body weight, mean±SD (kg)	59.72±9.03
K-L score, mean±SD (stage)	1.71±0.67
ROM, mean±SD (°)	
Extension	–1.77±3.25
Flexion	129.61±13.52
VAS score, mean±SD	48.00±23.85
JKOM score, mean±SD	32.74±13.93

n=number of patients, SD=standard deviation, K-L=Kellgren-Lawrence, ROM=range of motion, VAS=visual analog scale, JKOM=Japanese Knee Osteoarthritis Measure.

**Table2:** Correlations (ρ) between US findings and K-L score, ROM, VAS score, and JKOM score

	Medial meniscal protrusion (mm)			Synovial membrane thickness (mm)	Femoral osteophyte (mm)
	Extension	Flexion	Loading		
Mean±SD	7.44±3.42	9.00±3.64	8.52±3.94	4.95±1.45	4.00±1.28
K-L score	0.5962	0.6597	0.6276	ns	ns
ROM					
Extension	ns	ns	ns	ns	ns
Flexion	0.4410	0.3994	0.5810	ns	ns
VAS score	ns	ns	ns	ns	ns
JKOM score					
Total	ns	ns	ns	0.3723	ns
Pain and stiffness	ns	ns	ns	ns	ns
State of daily life	ns	ns	ns	ns	ns
Usual activity	ns	ns	ns	0.3590	ns
Influence of health	ns	ns	ns	ns	ns

ρ=Spearman correlation coefficient, US=ultrasound, K-L=Kellgren-Lawrence, ROM=range of motion, VAS=visual analogue scale, JKOM=Japanese Knee Osteoarthritis Measure, SD=standard deviation, ns=not significant. P<0.05 was considered statistically significant.
